# Retrobulbar chondrosarcoma in a dog

**Published:** 2014-04-30

**Authors:** M. Ralić, J. Vasić, M. Jovanović, B. Cameron

**Affiliations:** 1 *Veterinary surgeon, Kumanovo, 1300, Republic of Macedonia*; 2 *Department of Surgery, Orthopaedics and Ophthalmology, Faculty of Veterinary Medicine, University of Belgrade, 11000 Belgrade, Serbia*; 3 *Department of Pathomorphology, Faculty of Veterinary Medicine, University of Belgrade, 11000 Belgrade, Serbia*; 4 *Veterinary surgeon, Hague, The Netherlands*

**Keywords:** Chondrosarcoma, Computed tomography, Exophthalmos

## Abstract

This paper presents a review of a dog, with a retrobulbar chondrosarcoma, which was admitted for surgery for visible changes in his eye during inspection. Orbital neoplasia in dogs may be primary and secondary. Sixty percent of orbital neoplasia in dogs are primary, ninety percent of which are malignant. Retrobulbar neoplasms are rare and in their early stage represent a diagnostic challenge. Chondrosarcoma of the skull is a slow-progressing malignant disease which occurs locally, aggressive with invasion into the surrounding tissues. Dogs with chondrosarcoma of the skull have life expectancy between 210 and 580 days - in our case it was 180 days - after the first alterations on the eye of the dog occurred.

## Introduction

Chondrosarcoma (CS) is a malignant tumour of bones in animals and humans. This neoplasm corresponds to 5 - 10% of all the primary bone tumours in dogs. Primary CS may occur on skeletal system and then it is called central or medular CS. When CS is located on periosteum, it is called peripheral CS (Withrow and Vail, 2007).

### Case Details

Eight years and seven months old male Cocker Spaniel was admitted to the surgery for presence of a thick mucus discharge and changes on the cornea of his left eye. The dog apparently did not have any other health issues. During the clinical examination, the dog was fit, in a good mood and there were no changes in regional lymph nodes. The changes on the left eye were visible: presence of the dried mucus discharge and clingy hair on the upper eyelid and medial canthus.

Due to protrusion of the eye bulb, a pronounced exophthalmos of the left eye was clearly perceptible. The upper lateral quadrant of the cornea was blurry. There were no visible changes in the right eye.

The methods used during the first examination of the eye were: the fluorescein eye stain test, the Schirmer I tear test, measurement of the intraocular pressure and the Jones’ test for patency of nasolacrimal duct. As the process progressed, the tumour was sampled for histopathological examination and computed tomography of the skull was also performed in the follow-up examinations.

The intraocular pressure was measured by the Schiotz method, with prior application of anaesthetic (proparacain) to the eye. The solution of fluorescein was used for staining the cornea and testing the patency of the nasolacrimal duct. The computed tomography of the skull was performed by the third-generation device GE (9800-8800), under sedation using Domitor.

**Fig. 1:**
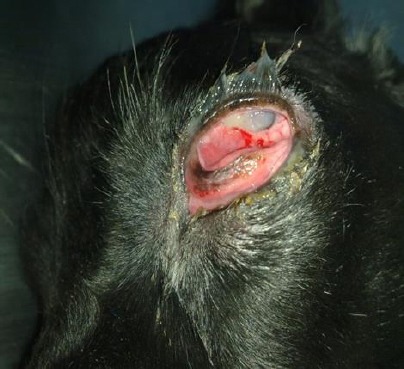
Dried mucous secretion on the eye lids, hyperaemia, oedema of the conjunctiva with protrusion of the third eye lid and keratitis (one month after the first examination).

The inspection revealed changes on the auxiliary parts of the left eye and presence of dried mucus discharge. Alopecia was not noticed on the lids of the left eye. The third eyelid protruded out of its place covering half of the eye bulb (Fig. 1).

The dog did not react painfully to retropulsion of the eye bulb, which was performed due to its enlargement. There was no counter effect from the bottom of the orbit of the affected eye during the retropulsion. The Schirmer I test results for the right and the left eye were 15 mm and 6 mm, respectively. There was a superficial whitish blur in the upper medial quadrant of the left eye cornea.

The fluorescein stain test was positive, which indicated alteration of the superficial layer of the cornea. The Jones’ test was positive for both nasolacrimal ducts. The changes on the iris and on the anterior chamber were not noticed during the ophthalmoscopy. Examination of the posterior segment of the eye and the lens of the left eye was performed after application of the Midriacil 1% drops to the eye. A central Y nuclear sclerosis was noticed in the lens.

There were no anatomic deviations on the fundus, both on the tapetal and non-tapetal part of the retina. A slight swelling of the optic papilla was observable. The mean values of the intraocular pressure of the right eye and the left eye were 22.38 mmHg and 10.24 mmHg, respectively.

The findings of the examination, absence of tears, moderate exophthalmos and slight protrusion of the third eye lid were basis for establishing the working diagnosis of keratoconjunctivitis sicca of the left eye, so further regular follow-ups were advised. The therapy for dry eye (artificial tears) and systemic antibiotic therapy (Cephalexin tabl. 15 mg/kg) were administered to the dog.

**Fig. 2:**
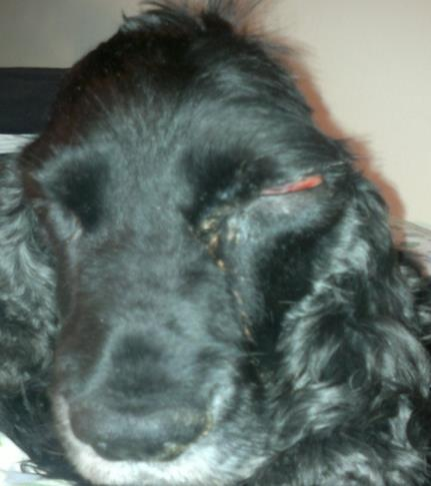
Lateral transposition of the left bulb and impossibility of retropulsion of the left eye to the orbit. Dried secretion on the left nostril.

A month later, the next examination revealed significant deterioration of the clinical signs on the surface of the cornea and significant enlargement of the eye bulb (Fig. 2).

The position of the left eye bulb indicated upward lateral strabismus. The third eye lid was everted and the conjunctiva was swollen. When performed again, the Jones’ test resulted with absence of staining in the left nostril which indicated obstruction of the left nasolacrimal duct.

The owner reported that the dog sniffles discharging a thick secretion from the left nostril and has swallowing problems. Computed tomography of the skull with prior application of sedative Domitor followed the examination of the mouth cavity, which revealed an obvious tumoural mass behind the last upper molar on the left side.

**Fig. 3:**
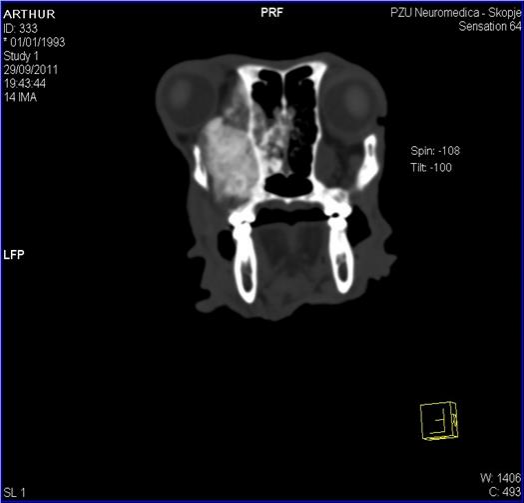
Computed tomography of the skull. The left eye bulb protrusion, presence of the tumoural mass in the very base of the orbit with osteolysis of the nasal septum and presence of the neoplasm in the left nasal duct.

The scan was performed in dorsolateral position (Fig. 3).

**Fig. 4:**
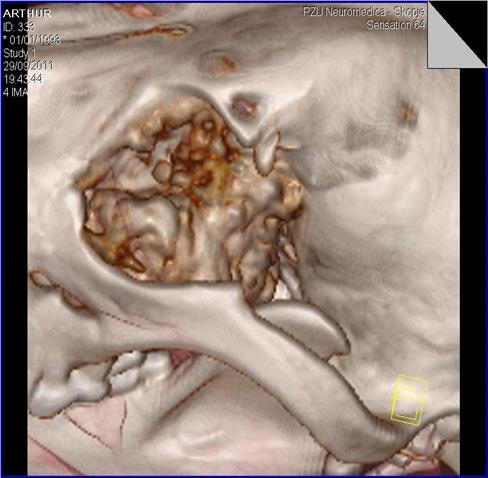
Reconsturction: neoplasia the bottom of the orbit.

**Fig. 5:**
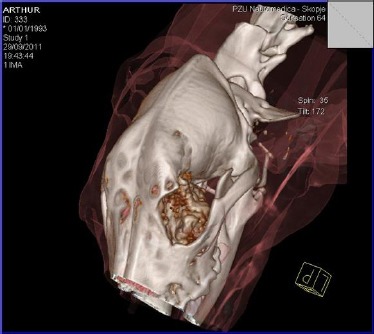
Reconstruction: the neoplasia affects the bottom of the orbit and osteolysis of part of the orbit.

The images clearly showed presence of the neoplasia in the region of the medial orbit, osteolysis of the nasal septum and presence of the tumoural mass in the left nasal duct (Fig. 4 and 5).

**Fig. 6:**
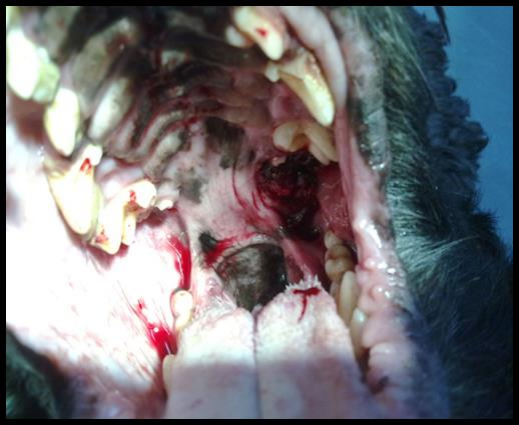
Position of the prominent part of the tumour behind the last molar of the maxilla.

The excisional biopsy of the neoplasm in the mouth cavity with prior application of xylocain 2% spray was performed after the scan (Fig. 6).

The system therapy including antibiotic and NSAID – antibiotic Cephalexin at a dose of 15 mg/kg p/o and Movalis tablets at a dose of 1.7 mg/kg BW per day – was applied, as the dog obviously suffered from pain. The excised tumour was assessed morpho-pathologically.

**Fig. 7:**
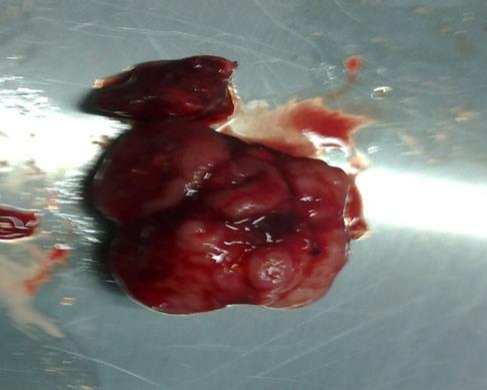
The part of the tumour removed for histopathological examination.

The tumoral mass was 3, 4×2, 5×1.8 cm in size and 25g in weight and hard in texture (Fig. 7).

**Fig. 8:**
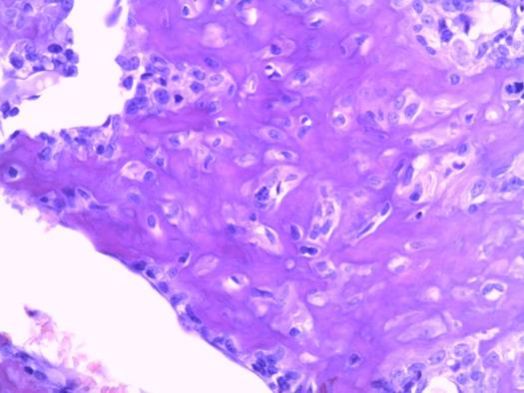
Depicted scattered pleomorphic binuclear chondrocytes. Clearly pronounced cytoplasm, due to the larger amount of glycogen. The isles of hyaline cartilage surrounded by non-defined mesenchymal cells (H&E ×300).

The preparation for histopathological examination was dyed with hematoxylin and eosin (H&E). Histopathological findings showed presence of pleomorphic cells with vesicular nucleus containing one or more nucleoli. The tumour cells produced the chondroid matrix and they were arranged, by their shape and size, in irregular groups which were separated from the surrounding tissue (Fig. 8).

Such histopathological finding indicated a highly differentiated mesenchymal sarcoma of the left eye with destruction of the nasal septum.

Since surgery and radical removal of the neoplasm, as well as application of chemotherapy, were not possible, the dog was given the medication for pain relief and the eye was treated locally with the antibiotic drops (Tobramicin 4×1). Three months after the final diagnosis was established, the dog died.

## Discussion

Primary neoplasia of the orbit can arise from any tissue: epithelial, vascular, nervous or connective tissue. Secondary orbital neoplasia arises from the nasal structures, sinuses or cranial cavities of the skull and rarely as distant metastasis. Meningioma, lymphosarcoma, adenocarcinoma, fibrosarcoma, myxoma, glioma, squamous cell carcinoma (SCC) and rabdomyosarcoma are among the numerous neoplasia of the orbit in dogs.

In dogs, 90% of orbital tumors are malignant. CS occurs on pelvis, facial bones, ribs, vertebrae and scapula as a non-painful tumoural mass (Bostock and Owen, 1975). The occurrence in trachea, lungs, omentum, mammary gland, heart valves, aorta, tongue, root of the penis and the penile urethra was also reported (Kim *et al.*, 2007).

CS is a malignant tumour cells which produce cartilaginous matrix. It develops from the cartilage structures of the bones and can also develop out of the tissues where cartilage normally does not exist. CS of the skull rarely occurs and most often, in 28.8% cases, it can be found in the nasal cavity, 9% of which is on the facial bones (maxilla, mandible and orbit) (Kim *et al.*, 2007).

Due to impossibility of the eye closure, the cornea gets dry and keratitis, conjunctivitis, myosis and eccentric pupil can be observed. The eye affected by CS of the orbit is non-functional, and due to destruction of the nasal septum and expansion of the tumor, a thick nasal discharge, difficult breathing and nasal stridor are also present.

Besides the clinical signs, ultrasonography of the eye and the orbit, fine-needle aspiration biopsy (FNA), plain radiography of the skull, computed tomography and magnetic resonance imaging (MRI) are also the methods used in diagnosing CS of the orbit. Regarding the differential diagnosis, CS should be distinguished from non-neoplastic inflammatory orbital granuloma, cellulitis and abscesses and from myositis of extraocular muscles and masticatory muscles.

The main symptom in the dogs with inflammatory processes in the region of the orbit is presence of a strong pain during mouth opening. The dogs with inflammatory processes in the region of the orbit feel pain during mouth opening (Withrow and Vail, 2007).

The following six parameters are being used in CT scan and in diagnosis of tumours of the nose, the superior sinuses and the orbit:

1. Bilateral expansion of the tumour in basal and paranasal sinuses.
2. Destruction of the nasal bone and finding of tumoural mass on nasal surface and in the facial area.
3. Presence of the tumour in the mouth cavity with hard palate destruction.
4. Presence of the tumour in the orbit and lateral pressure on the bulb.
5. The frontal sinus involvement.
6. The brain involvement and destruction of the cranium, expansion of the tumour into the brain (Kondo *et al.*, 2008).

Radiography reveals presence of the irregular and often blurred areas permeated by calcification. Atypical cartilaginous tissue with metachromatic basal material can be observed in the histopathological finding. Some cells and some groups of cells are binuclear. Especially characteristic are the big atypical cells with large “swollen-plump” nuclei (Zlateva and Milenkov, 1983). All degrees of differentiation occur; benign tumours have an abundant cartilaginous matrix whilst malignant tumours contain closely packed cells with plump, frequently multiple nuclei. Mitotic figures are common in the more malignant tumours (Bostock and Owen, 1975).

Sometimes, calcifications and ossifications can be found in the cartilaginous tissue of the tumour. Treatment for CS of the skull is directly dependent on its localization and the degree of invasiveness towards the surrounding tissue. As it is a malignant tumour, the treatment of choice, if it is possible, would be the surgery.

Orbitotomy with resection of zygomatic arch is recommended. If the tumoural mass is small, a transconjuctival approach to the tumour is also possible (Maggs *et al.*, 2008). In the case of the surgery, the operation is difficult because differentiation between the normal and the tumoural tissue is not easy.

For this reason, it can happen that the neoplasm is removed incompletely, which causes consequent recurrences (Gelatt and Gelatt, 2011). In the case that the tumour invaded the brain, the mouth cavity, or the orbit, radiotherapy – either as monotherapy or combined with chemotherapy – and sometimes (as it was in our case) even only the antibiotic and NSAID treatment are recommended before the surgical treatment.

Prognosis for the orbital tumours is poor since the surgical treatment does not solve the issue of recurrences. The dogs with orbital osteolysis (as in our case) have even poorer prognosis. In the case of radical surgery (orbitectomy), the owners are reluctant to accept the fact that their pet is going to have the postoperative facial deformity. The studies suggest that surgical treatment of CS and chemotherapy can prolong life of the dog for six months (Gelatt and Gelatt, 2011). The average life expectancy for dogs with CS that also invaded the nasal cavity is between 210 and 580 days, with treatment provided (rhinotomy with radiotherapy) (Withrow and Vail, 2007). The low-grade chodrosarcoma can grow for a long time during several years without showing a tendency to metastasize, while the fast-growing CS metastasizes to lungs, kidneys, liver and heart (Withrow and Vail, 2007).

CS of the orbit is a rare, slow-progressing malignant disease in dogs and is extremely destructive to surrounding tissues. In the cases of clinically diagnosed CS in dogs, the dogs were in the age of about eight years. On the computed tomography scans one can observe a large, irregularly shaped mass with destruction of the orbital bones and mineralisation of the tumoural matrix. There is also compression of the local tissue, the muscles of the eye bulb and dislocation of the eye bulb from the orbit.

Due to the difficult approach to the tumour and the pronounced destruction of the surrounding tissues, it is difficult to perform the surgery. Recurrences are very frequent and the life expectancy in dogs is less than a year after the accurate diagnosis is established. Due to the severe pain, it is necessary to apply the potent NSAID and opioid analgesics as well as the supportive therapy including vitamins and intravenous solutions to bring some relief to the dog.
